# Motion energy analysis during speech tasks in medication-naïve individuals with at-risk mental states for psychosis

**DOI:** 10.1038/s41537-022-00283-3

**Published:** 2022-09-16

**Authors:** Ana Caroline Lopes-Rocha, Cheryl Mary Corcoran, Julio Cesar Andrade, Leonardo Peroni, Natalia Mansur Haddad, Lucas Hortêncio, Mauricio Henriques Serpa, Martinus Theodorus van de Bilt, Wagner Farid Gattaz, Alexandre Andrade Loch

**Affiliations:** 1 Laboratorio de Neurociencias (LIM 27), Instituto de Psiquiatria, Hospital das Clinicas HCFMUSP, Faculdade de Medicina, Universidade de Sao Paulo, Sao Paulo, SP, BR Brazil; 2 Department of Psychiatry, Icahn School of Medicine at Mount Sinai, New York, NY 10029 USA; 3 Laboratorio de Neuroimagem em Psiquiatria (LIM 21), Instituto de Psiquiatria, Hospital das Clinicas HCFMUSP, Faculdade de Medicina, Universidade de Sao Paulo, Sao Paulo, SP, BR Brazil; 4 Instituto Nacional de Biomarcadores em Neuropsiquiatria (INBION), Conselho Nacional de Desenvolvimento Cientifico e Tecnológico, Sao Paulo, Brazil

**Keywords:** Psychosis, Schizophrenia

## Abstract

Movement abnormalities are commonly observed in schizophrenia and at-risk mental states (ARMS) for psychosis. They are usually detected with clinical interviews, such that automated analysis would enhance assessment. Our aim was to use motion energy analysis (MEA) to assess movement during free-speech videos in ARMS and control individuals, and to investigate associations between movement metrics and negative and positive symptoms. Thirty-two medication-naïve ARMS and forty-six healthy control individuals were filmed during speech tasks. Footages were analyzed using MEA software, which assesses movement by differences in pixels frame-by-frame. Two regions of interest were defined—head and torso—and mean amplitude, frequency, and coefficient of variability of movements for them were obtained. These metrics were correlated with the Structured Interview for Prodromal Syndromes (SIPS) symptoms, and with the risk of conversion to psychosis—inferred with the SIPS risk calculator. ARMS individuals had significantly lower mean amplitude of head movement and higher coefficients of movement variability for both head and torso, compared to controls. Higher coefficient of variability was related to higher risk of conversion. Negative correlations were seen between frequency of movement and most SIPS negative symptoms. All positive symptoms were correlated with at least one movement variable. Movement abnormalities could be automatically detected in medication-naïve ARMS subjects by means of a motion energy analysis software. Significant associations of movement metrics with symptoms were found, supporting the importance of movement analysis in ARMS. This could be a potentially important tool for early diagnosis, intervention, and outcome prediction.

## Introduction

Movement abnormalities are widely observed in schizophrenia spectrum disorders. They are associated with worse outcomes and a declining course of illness, and they are also present in early stages of these disorders^1–4^. In fact, neuromotor precursors of schizophrenia can be traced back to childhood^5^. An analysis of brief videotape footage of children eating lunch suggested that observed movement anomalies were able to discriminate among those children who later developed schizophrenia and those who did not^6^. Accordingly, it is useful to assess movement in young people at risk for schizophrenia.

In this sense, subjects at-risk mental states (ARMS) for psychosis present subtle changes in perception, belief and thought^7,8^, as well as incipient negative symptoms such as avolition, anhedonia, and blunted affect^9,10^. Blunted affect, for instance, is characterized by impairment in expressive gestures, spontaneous movements, eye contact, facial expression, and others^11–13^. This affective disturbance is reflected in subjects’ movements while in social interactions, resulting in the reduced overall quantity of movements, asynchronous gestures and poor social skills^14^. A recent meta-analysis described a significant association between blunted affect and poor social functioning in ARMS subjects^15^. Movement in schizophrenia spectrum disorders is also impacted by motor abnormalities, including hyperkinetic and hypokinetic abnormal involuntary movements (AIMs), and catatonia, resulting in an increased variability of movements^16^. Therefore, during communication, these may be expressed by more erratic and variable/irregular gestures, further impairing social skills^17^. Recent studies have documented abnormal involuntary movements in ARMS subjects too, showing brain functional and structural correlates^1^.

As such, investigations carried out by Mittal’s research group have shown the importance of movement analysis in at-risk individuals. Among their findings, higher AIMs scores^18,19^, motor slowing^20^, mismatch between gestures and speech^2^, more gestures made during pauses in speech^2^, increased postural sway^21^, and less gesticulation in some gestural categories^22^ were seen in these subjects compared to healthy individuals. In addition to the expected association between these abnormalities and negative symptoms, their studies also found association with positive symptoms. Movement abnormalities were positively associated with positive symptom severity^19,23^ and increased upper-body abnormalities were correlated with increasing positive symptom severity over one year of follow-up^24^.

However, in most of these works movement abnormalities were observed by trained raters. An automated paradigm capable of assessing movement would be of great value, as it could add features to the potential predictive value of ARMS status by capturing subtle changes beyond expert ratings^25,26^. Also, observer-based methods in general require more than one rater given the time necessary to complete the analysis and this whole process is time-consuming concerning training, validation, and analysis—a time that could be greatly reduced by automated methods. At last, automated analysis is also free of rater bias^26–28^.

Automated analysis was previously performed, for example, through movement sensors coupled to the region of interest to be analyzed. Leask et al. analyzed the head movement of 11 schizophrenia patients, who were prescribed antipsychotic medications—and found decreased amplitude and velocity of movement^29^. Altorfer et al. analyzed 23 first episode psychotic patients using a system that measures the coordinates of ultrasonic transducers positioned in the regions of interest^30^ and found a reduced amplitude and frequency of head movement, that was not accounted for by medication status^31^. Nevertheless, here we present data using video analysis, which is easier to administer and less intrusive, specifically Motion Energy Analysis (MEA)^32^.

MEA is a software program based on frame-differencing methods that evaluate differences in greyscale pixels frame by frame^32^^,33^. That is, when the camera is fixed and the person being filmed moves, there is a change in pixel density, which is quantified as movement. This software is often used to verify the nonverbal synchrony—the coordination of movements—between patients and therapists, which impacts the psychotherapy outcome through self-reported quality of the relationship and further variables of the therapy process^34,35^. It has already been used to assess movement abnormalities in a few samples of schizophrenia^33,36^ and ARMS individuals^25^. In Kupper et al.’s MEA study, 27 schizophrenia patients and 27 controls underwent role-play tests of 14 scenes. A slight reduction in head and body movement was observed in schizophrenia patients compared to controls^36^. Also, they found a correlation between reduced movement and negative symptoms—mainly emotional withdrawal, as measured by the Positive and Negative Syndrome Scale (PANSS)^37^. In a study of 54 ARMS patients, Dean et al. applied MEA analysis to video from the first 15 min of the Structured Interview for Psychosis-Risk Syndromes (SIPS), finding no difference in head movement and no correlation symptoms, but did find increased total body movement and speed^25^. Of note, both studies did not analyze videos collected during free-speech tasks, which allows the assessment of movement during unstructured, spontaneous interactions.

Similarly to the protocol used by Dean et al.^25^, the present study aimed to investigate differences in torso and head movements between 32 individuals identified as “at risk mental state” for psychosis, as compared to 46 healthy controls. Free speech was elicited and videorecorded during SIPS’ subject overview (plus participant talking about his relationship to parents during childhood—SO) and solicitation of dream and memory reports (MR). Based on the findings of Kupper et al.^36^, Leask et al.^29^, and Altorfer et al.^31^, we hypothesized that compared to healthy controls, (1) ARMS individuals would have lower mean energy motion/lower frequency of movement, and a higher variability of movements. Based on Kupper et al.^36^ and Mittal et al.^19,23,24^ studies, we further hypothesized that (2) movement metrics would be associated with negative and positive symptoms in ARMS subjects.

## Results

The ARMS group and healthy controls were similar in sociodemographics (Table 1). They were between the ages of 18 and 36 years old (mean (SD) = 27.6 (4) years) with more than two-thirds female (69.2%). All ARMS and control participants were medication-free at the time of testing. The scores for SIPS negative and positive symptoms are available in Table 1, as well as the percentage that met APSS and GRD criteria.

**Table 1:** Sociodemographic and SIPS symptoms of ARMS and Control groups.

		ARMS	Control	Statistic	ρ
Age (years)^a^		27.0 ± 3.8	28.5 ± 4.3	t(75) = −1.51	0.13
Gender^b^	Female	22 (68.8)	32 (69.6)	χ² (1.78)=0.0059	0.94
	Male	10 (31.3)	14 (30.4)		
Education (years)^b^	0-6	13 (37.1)	12 (30)	*U*(75) = 682	1.0
	7-8	18 (51.4)	26 (65)		
	9-10	4 (11.4)	2 (5)		
Negative Symptoms^a^	Total	6.81 ± 4.30	4.35 ± 3.91	*U*(75) = 481	0.009
	N1	1.09 ± 1.42	0.98 ± 1.11	–	–
	N2	1.50 ± 1.27	1.04 ± 1.09	–	–
	N3	0.78 ± 0.83	0.41 ± 0.69	–	-
	N4	1.22 ± 1.01	0.59 ± 0.77	–	–
	N5	1.34 ± 1.41	0.54 ± 0.89	–	–
	N6	0.87 ± 1.10	0.78 ± 1.15	–	–
Positive Symptoms^a^	Total	9.53 ± 3.16	4.24 ± 2.45	*U*(75) = 150	<0.001
	P1	2.44 ± 1.16	0.96 ± 0.66	–	–
	P2	2.22 ± 1.16	1.26 ± 0.85	–	–
	P3	0.91 ± 1.03	0.17 ± 0.38	–	–
	P4	3.16 ± 1.05	1.22 ± 1.03	–	–
	P5	0.81 ± 0.96	0.63 ± 0.71	–	–
Type of risk syndrome	APSS	32 (100)	–		
	GRD	3 (9.4)	–		

^a^Mean ± SD.

^b^*N* (%).

**Fig. 1:**
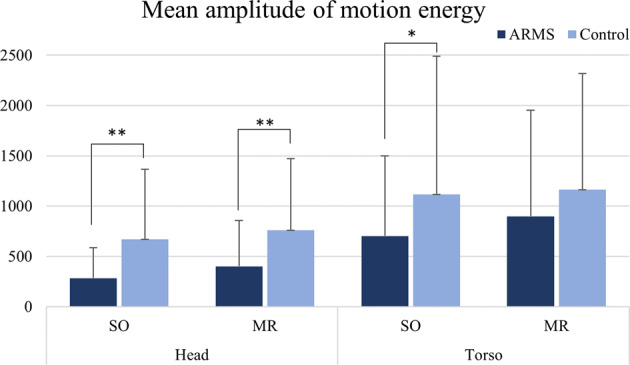
Mean amplitude of motion energy analysis for both regions of interest in subject overview (SO) and memory report (MR) videos. *p < 0.05: significant using independent-samples *T* tests. **p < 0.0125: significant after Bonferroni correction for multiple comparisons. Data expressed as mean amplitude of motion across the entire video analysis ± standard deviation (s.d.).

Concerning our first hypothesis of movement differences between ARMS and controls, head’s mean amplitude of movement was significantly lower in ARMS compared to controls in both videos (SO: *U* = 422, *p* < 0.001, *d* = 0.414; MR: *U* = 418, *p* = 0.009, *d* = 0.323) (significant after Bonferroni correction, *p* < 0.0125) (Fig. 1). For the torso ROI, a lower mean amplitude for ARMS was found for the SO task (*U* = 539, *p* = 0.031, *d* = 0.251), however significance did not survive Bonferroni correction for multiple comparisons.

**Fig. 2:**
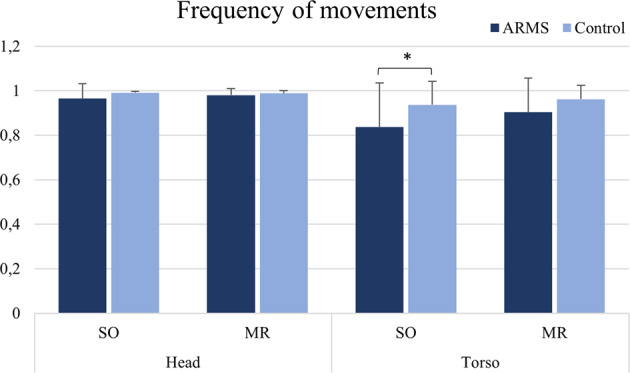
Frequency of movements during motion energy analysis for both regions of interest in subject overview (SO) and memory report (MR) videos. *p < 0.05: significant using independent-samples *T* tests. It did not survive Bonferroni correction for multiple comparisons. Data expressed as mean of non-zero sum of frames divided by the total number of frames ± standard deviation (s.d.).

For frequency of movements, torso’s mean values were significant lower in ARMS as compared to controls in the SO video (*U* = 504, *p* = 0.013, *d* = 0.300), but that did not survive Bonferroni correction (*p* > 0.0125) (Fig. 2).

**Fig. 3:**
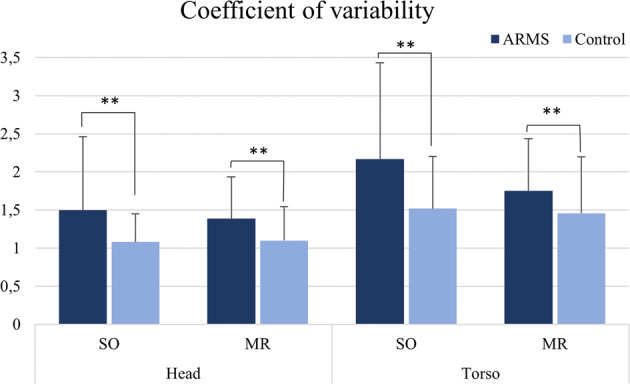
Coefficient of variability during motion energy analysis for both regions of interest in subject overview (SO) and memory report (MR) videos. **p < 0.0125: significant after Bonferroni correction for multiple comparisons. Data expressed as mean of standard deviation of amplitude of motion divided by mean amplitude of motion across the entire video analysis ± standard deviation (s.d.).

As for variability of movements, ARMS significantly differed from controls in all ROIs and videos, even after Bonferroni correction (*p* < 0.0125). Higher scores were observed for ARMS as compared to controls both for the head (SO video: *U* = 477, *p* = 0.006, *d* = 0.338; MR video: *U* = 347, *p* < 0.001, *d* = 0.443) and the torso ROI (SO video: *U* = 490, *p* = 0.009, *d* = 0.319; MR video: *U* = 412, *p* = 0.007, *d* = 0.339) (Fig. 3).

Concerning our second hypothesis of movement variables being linked to negative symptoms, frequency of movements was significantly correlated with negative symptoms, but results did not survive Bonferroni correction (*p* < 0.004) (Table 2). In MR video, head movement frequency was negatively correlated with N1, N3 and N4 (τ = −0.180, *p* = 0.046, τ = −0.203, *p* = 0.030, and τ = −0.185, *p* = 0.044, respectively), and torso frequency of movement was negatively correlated with N3 (τ = −0.199, *p* = 0.033). Likewise, N5 also showed a negative correlation with head’s amplitude of movement (SO video: τ = −0.186, *p* = 0.034, and MR video: τ = −0.202, *p* = 0.027) and frequency (SO video: τ = −0.174, *p* = 0.048).

**Table 2:** Correlation between movement variables and symptoms collected during SIPS.

Video	ROI	Measure		N1	N2	N3	N4	N5	P1	P2	P3	P4	P5	*P*_total_
SO	Head	Amplitude	Tau-B	0.102	0.050	−0.136	−0.013	−0.186*	−0.230**	−0.196**	−0.220*	−0.297***	−0.236**	−0.293***
			*p-*value	0.241	0.560	0.134	0.884	0.034	0.007	0.023	0.015	<0.001	0.009	<0.001
		Frequency	Tau-B	−0.022	0.139	−0.104	−0.004	−0.174*	−0.140	−0.173*	−0.189*	−0.257***	−0.081	−0.216**
			*p-*value	0.801	0.107	0.251	0.966	0.048	0.103	0.044	0.037	0.002	0.368	0.007
		CV	Tau-B	−0.060	0.008	0.093	0.041	0.116	0.211*	0.110	0.079	0.272***	0.161	0.216**
			*p-*value	0.491	0.931	0.304	0.640	0.185	0.014	0.198	0.381	0.001	0.073	0.007
	Torso	Amplitude	Tau-B	−0.010	−0.006	−0.127	0.002	−0.028	−0.170*	−0.071	−0.094	−0.215*	−0.085	−0.153
			*p-*value	0.913	0.945	0.162	0.981	0.754	0.048	0.407	0.300	0.011	0.343	0.056
		Frequency	Tau-B	0.072	0.166	−0.059	0.044	−0.039	−0.172*	−0.045	−0.152	−0.254**	−0.013	−0.160*
			*p-*value	0.412	0.055	0.511	0.620	0.654	0.045	0.597	0.094	0.003	0.885	0.045
		CV	Tau-B	0.024	−0.019	0.147	0.055	−0.006	0.188*	0.052	0.057	0.225**	−0.010	0.144
			*p-*value	0.780	0.822	0.105	0.536	0.950	0.029	0.541	0.529	0.008	0.915	0.072
MR	Head	Amplitude	Tau-B	−0.022	0.008	−0.135	−0.070	−0.202*	−0.220*	−0.194*	−0.167	−0.261***	−0.268***	−0.285***
			*p-*value	0.804	0.927	0.148	0.446	0.027	0.013	0.030	0.076	0.003	0.004	<0.001
		Frequency	Tau-B	−0.180*	−0.027	−0.203*	−0.185*	−0.113	0.019	−0.137	−0.098	−0.088	−0.101	−0.089
			*p-*value	0.046	0.761	0.030	0.044	0.216	0.832	0.125	0.298	0.311	0.278	0.283
		CV	Tau-B	0.048	0.054	0.018	0.030	0.102	0.233**	0.140	0.142	0.235**	0.138	0.252***
			*p-*value	0.590	0.543	0.847	0.746	0.263	0.009	0.115	0.131	0.007	0.136	0.002
	Torso	Amplitude	Tau-B	−0.094	0.003	−0.082	0.026	−0.052	−0.067	−0.026	0.013	−0.133	−0.136	−0.085
			*p-*value	0.296	0.976	0.382	0.778	0.567	0.449	0.770	0.890	0.128	0.142	0.305
		Frequency	Tau-B	−0.156	0.104	−0.199*	−0.122	−0.043	−0.227*	−0.061	−0.053	−0.222*	−0.145	−0.213*
			*p-*value	0.084	0.244	0.033	0.181	0.634	0.010	0.492	0.576	0.011	0.117	0.010
		CV	Tau-B	0.171	0.080	0.088	0.050	−0.006	0.163	−0.055	−0.030	0.218*	0.051	0.118
			*p-*value	0.057	0.372	0.347	0.587	0.944	0.066	0.538	0.750	0.012	0.582	0.154

*p < 0.05, **p < 0.01: Significant by Kendall’s tau-B correlation coefficient. ***p < 0.004: Significant Bonferroni correction for multiple comparisons.

**Fig. 4:**
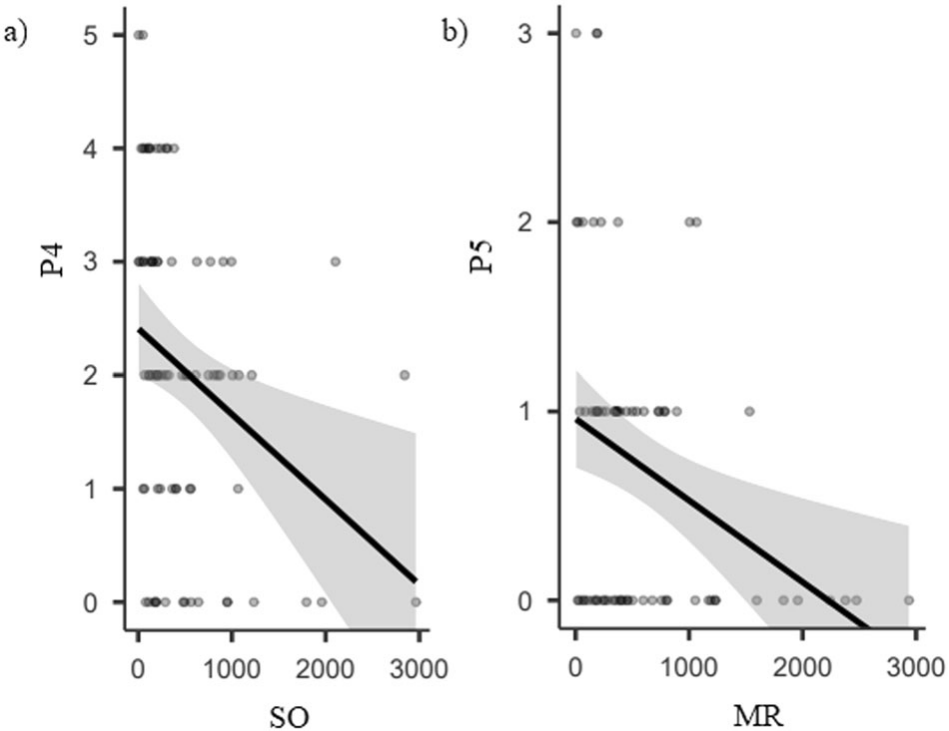
Examples of correlations scatter plots between positive symptoms and head mean amplitude in subject overview (SO) and memory report (MR) videos. Negative correlation between head mean amplitude and **a** perceptual abnormalities/hallucinations (P4) in SO and **b** disorganized communication (P5) in MR video were significant even after correction for multiple parisons (*p* < 0.004). Scores for each symptoms are expressed in *Y*-axis, while the *X*-axis represents the head mean amplitude (mean amplitude of motion across the entire video analysis) collected by motion energy analysis16.

For positive symptoms, we found a significant correlation between every symptom and at least one movement variable, as well as the sum of P scoring. These correlations reiterate the findings above: the higher the positive symptom the lower the movement frequency and amplitude (p-values varying from 0.048 to <0.001), and the higher the positive symptom the higher the movement variability (*p*-values ranging from 0.029 to 0.001). Of note, all positive (P) symptoms were significantly related to all measures of head amplitude (with one exception out of 10), and total P score showed a significantly correlation with this measure (*p* < 0.001 in both videos). After Bonferroni correction (*p* < 0.004), head measures in SO, torso frequency in SO and head amplitude in MR remained significantly correlated with P4 symptom as well as head amplitude in MR with P5, total *P* with head amplitude in both video and with head coefficient of variability in MR (Tables 2). Scatter plots to exemplify some correlations are represented in Fig. 4 and summary of findings are available in Table 3.

**Table 3:** Summary of findings.

Movement feature	ROI	Association with SIPS symptoms	
		Negative	Positive
Amplitude	Head	N5	P1, P2, P3, P4*, P5*
	Torso	–	P3
Frequency	Head	N1, N3, N4	P1, P2, P3, P5
	Torso	N3	P1, P4
Variability	Head	–	P1, P4*
	Torso	–	P1, P4

*Significant after Bonferroni correction for multiple comparisons.

Correlating the risk of conversion provided by the SIPS-RC calculator with movement variables, a positive correlation was found for mean amplitude of head (τ = −0.172, *p* = 0.027) and frequency of torso movements (τ = −0.187, *p* = 0.018) in MR video. These results did not survive Bonferroni correction (*p* > 0.00625). A significant correlation between conversion and head’s coefficient of variability in MR was found (τ = 0.238, *p* = 0.004)(significant after Bonferroni correction, *p* < 0.0125).

Movement variables were not significantly related to either sex or age (data not shown).

## Discussion

In this study of Brazilian medication-naïve individuals, we found decreased head’s amplitude of movement in youths with an at-risk mental state (ARMS), as compared to healthy controls. This is consistent with findings by Kupper et al. in schizophrenia patients, who also used MEA analyses of video, but obtained the footage during role-play^36^. Furthermore, we found a higher coefficient of movement variance in ARMS individuals in both head and torso. Our findings are in contrast to what was found by Dean et al., who also used MEA to analyze movement in ARMS subjects, and may be accounted for by differences in the video recording context^25^. Our replication of findings by Kupper et al.^36^ supports that abnormal movements are unlikely to be associated with the prescription of antipsychotic medications, as our cohort was medication-naïve, and also supports that these movement abnormalities can be identified in earlier stages of psychotic disorder. Also consistent with Kupper’s and Mittal’s findings, movement metrics were further associated with negative and positive symptoms^19,23,24^. As ARMS individuals present movement abnormalities before conversion to a psychiatric diagnosis, investigating these disturbances can contribute to diagnosis and intervention in the early stages. Accordingly, our results also showed that the risk of conversion is higher in those subjects with higher variability of movements.

The significant reduction in head amplitude of movement—and a probable reduction in torso’s amplitude and frequency of movement—in our ARMS individuals is in line with the literature^36^. Given that MEA is a measure of how much the filmed person has moved, co-speech gestures, self-stimulation, postural sway, and other movements contribute to the measure. As gesture impairments are seen both in schizophrenia^38^ and ARMS subjects^22^—as for example a reduction in beat movements of hands, rhythmic movements made together with the speech—we can speculate that they may have contributed to the reduction in our metrics^39^.

Our results, however, are different from those of Dean et al., who also used MEA analysis of video with ARMS subjects, analyzing the total amount of movement, mean amplitude, speed, and coefficient of variability^25^. Of note, they did not find any differences in head movement variables between ARMS and controls, but found increased—instead of decreased—total body movement in ARMS subjects. This may be related to the difference in the analyzed videos, as they recorded the participants during their structured interview. This may have elicited anxiety and restlessness in them, as has been seen in subjects with schizophrenia^40^. Other differences include the fact that their sample had higher rates on positive, negative, and disorganized symptoms, and that some participants in their study were on antipsychotic medication, which could have impacted their analysis, as suggested in the previous literature^36^.

Interestingly, our study found increased movement variability for both head and torso regions, meaning that ARMS subjects move less in general, but that when they do, they do it in a more erratic way. This could hypothetically be related to impaired motor function and postural sway. Several studies have shown that at-risk individuals have increased postural sway^41,42^ and motor abnormalities^18,19^, such as neurological soft signs (NSS)^43,44^. However, we did not include any specific abnormal involuntary movement measure or other instrumental measures and tasks for motor abnormalities. As such, this interpretation of the results should be faced with caution, and further investigation of this hypothesis should be conducted.

Kupper et al. showed an inverse correlation of body and head movement with negative symptoms, and of head movement with positive symptoms in schizophrenia, which support our findings^36^. In the present study, many negative symptoms indicated that higher symptom scores were associated with less frequency of movement. In the same way, decreased amplitude of movement was significantly related to higher positive symptoms. This result is also consistent with previous findings in adolescents at risk for psychotic disorders in which a dyskinesia scale was used. Facial movement abnormalities were associated with severity of both negative and positive symptoms, but for the upper body region the association was only seen for the negative symptoms^19^. Interestingly, facial movement abnormalities assessed over a follow-up period tend to have a constant correlation with positive symptoms while a decrease in the correlation with negative symptoms occurs. For the upper body region, the correlation with both symptoms tends to increase over time^24^.

At last, the associations seen here between movement features and symptoms provide a potentially important tool for the diagnosis and follow-up of ARMS individuals. Considering that increased postural sway predicts negative symptoms progression^21^, and that catatonic-like symptoms in first-episode drug-naïve patients predict a poor long-term psychosocial functioning^45^, movement abnormalities observed here could be a proxy for worse outcome.

Furthermore, literature shows that ARMS individuals who converted to psychosis exhibited more movement abnormalities than those who did not convert^23^, and therefore the mechanisms behind these abnormalities may play a role in disease pathophysiology^19,41^. In general, movement disturbances and cognitive deficits in ARMS are associated with cortico-striato-pallido-thalamic circuit irregularities^46^. Postural control deficits—such as postural sway—seems related to deficits in sensorimotor integration and cerebellar dysfunction;^41^ impaired gesture performance has been linked, for instance, with grey matter alterations in several regions^47^. As such, movement changes collected by the MEA may be the visible reflection of early biological changes that occur in the course of schizophrenia spectrum disorders. However, this underpinning should be investigated by further examination of such individuals—e.g. through imaging studies. At last, the risk of conversion in our study was related to higher variability of movements, showing the importance of analyzing such movement abnormalities. Nevertheless, this link is hypothetical and should also be further investigated in the future with follow-up data.

An important point to consider in our study is that we opted to perform two different speech tasks, both to verify their performance and aiming at a free and spontaneous speech of the participant in different moments of the clinical interview. Despite the presence of movement differences in both, more significant results were seen for the SO video—made at the beginning of the interview. It is possible that this result occurred because of the unstructured nature of the task, which allowed the person to express themselves in a freer way. However, this interpretation is speculative and needs further cross-protocols comparisons.

Another important point to consider for the present work is the cultural difference that can occur in studies that seek to assess the movement of individuals. Evidence suggests that there are cross-cultural differences in co-speech gestures, such as form-meaning associations, spatial cognition, language, and gestural pragmatics^48^. This study investigated movement energy specifically in Brazilian antipsychotic-naïve at-risk individuals and many of our results corroborate what is found in the literature. It would be of interest to compare Brazilian and American healthy controls to determine the role of culture in normal movement.

Our study has some limitations. First, given the cross-sectional design with a modest and heterogeneous sample, it is difficult to interpret null data. But despite being heterogeneous, our sample did not differ demographically between the groups analyzed and considering the use of a non-help-seeking sample, our sample size is similar to those from previous studies^49,50^. Second, the absence of clinical ratings of abnormal movements for estimations of construct or convergent validity. Third, our study lacks leg movement data. This occurred because our methodology was focused on a real-world scenario where usually the clinical setting is given by the patient sitting behind a table where the psychiatrist cannot access leg movements. Fourth, we have the limitations given by the software that, by keeping ROIs fixed, ends up preventing the division of the analysis of regions that transpose each other in many frames—such as hands and trunk. Finally, we did not specifically measure or isolate gesticulation, also because of the MEA method used. This may have contributed to some of the variance in metrics of torso movement in our study, but this needs to be addressed in future studies. It is important to note that data on follow-up of this cohort is still being collected and we believe that they will be important to understand mechanisms behind early psychosis and indicators of conversion in movement itself.

In sum, we used an automated algorithm—MEA analysis of videos—to demonstrate differences in motor behavior between ARMS and control individuals during free-speech tasks, replicating prior studies in the US. We now demonstrated these effects in a Brazilian cohort, who were, additionally, naïve to psychotropic medications. As this MEA analysis is easy to implement, requiring only video recording by a mobile phone, it provides an inexpensive and potentially scalable way to assess face and torso movement as part of screening efforts to detect young people at risk for psychosis. For future studies, larger samples would be enrolled to assess the generalizability of these findings across cultures, and its covariance with demographics and other features in the general population. Also, correlation with biological data would be useful to characterize mechanisms, important for identifying targets for preventive intervention.

## Methods

### Sample and procedures

This study is part of the Subclinical Symptoms and Prodromal Psychosis (SSAPP) Project, which consists of a population-based cohort study situated in São Paulo City, Brazil, involving over 2500 individuals aged 18–35 years. First, individuals were interviewed by telephone interview using the Prodromal Questionnaire-Brief version (PQ-16) and the Basic Symptoms scale (BS), following previously published screening procedures^51^. The PQ-16 is a shorter version of the original 92 items used in the Prodromal Questionnaire (PQ)^52^, which consists of a self-report questionnaire with 16 items to screen for ARMS of developing psychosis^53^. The BS is a criterion based on the basic symptoms of self-experienced disturbances in perception and cognition that are present in the initial manifestations of psychosis risk^51,54^.

Then, individuals with combined score >10 on the PQ-16 were called for a face-to-face interview at the Institute of Psychiatry, University of Sao Paulo, Brazil. They were assessed with the Structured Interview for Psychosis-Risk Syndromes (SIPS)^55^ for ARMS status, and with the Structured Interview for DSM-5 diagnosis (SCID-5)^56^. The SIPS is a structured diagnostic interview which diagnoses three prodromal syndromes for psychosis: the Brief Intermittent Psychotic Symptom syndrome (BIPS—experience of brief intermittent psychotic symptoms), the Genetic Risk and Deterioration syndrome (GRD—history of psychotic disorder in a first-degree relative or schizotypal personality, and a decline of 30% on the Global Assessment of Functioning Scale (GAF) in the past year) and the Attenuated Psychosis Syndrome (APS—presence of attenuated psychotic symptoms in the past year that are present at least once per week in the last month and have not reached a psychotic level)^57^. The SCID-5 is a semi-structured interview for the evaluation of DSM-5 disorders, including psychotic disorders^56^. Individuals who met criteria for other DSM disorders were not selected for any of the sample groups as well as individuals who used medications. After these interviews, 32 individuals were determined to meet criteria as ARMS status and 46 as healthy comparison subjects, all participants medication naïve.

### Elicitation of language and expression

Two protocols were applied, and audiovisual files collected by means of mobile phone positioned on a steady support, with participants sitting in front of the mobile phone. Informed consent was provided by all participants, and approval by the Institutional Review Board at the University of Sao Paulo. The first protocol consisted of SIPS subject overview, with the addition of an instruction to ask the subject to speak freely specifically about their childhood and relationship with their parents (Subject Overview—SO) and was conducted at the beginning of the interview. The second—performed at the end—was based on the paradigm of Mota^58,59^, consisting of requesting oral memory reports (MR): a recent dream, an old dream and short-term memory reports based on 3 positively affective pictures: a baby, a puppy and a dessert. When participants did not remember a dream, they were prompted to describe the prior day.

After collection, video was immediately stored in a secure cloud service and deleted from the mobile, where SO had an average duration of 6.13 ± 3.76 min and the MR of 3.73 ± 1.19 min. Protection was granted by means of current encryption protocols in the backend database and over the remote communications (SSL) according to Brazilian data protection compliance standards (Lei Geral de Proteção de Dados, LGPD; https://www.lgpdbrasil.com.br).

### Motion energy analysis

The motion energy analysis (MEA) was automatically obtained through the open-source software program based on frame-differencing methods^32,34^. The amount of movement can be evaluated in predefined regions of interest (ROI) and, for this study, due to the positioning of the individuals throughout the interview, two ROIs of interest were selected: the torso (for the assessment of upper body, hand and arms movements) and the head (Supplementary Fig 1). However, an intrinsic limitation of this method is that the ROIs are predefined, which means that if movements are made, for example, with a hand transposing the ROI of the head—such as when someone runs their hand through their hair—it will be quantified as movements performed within the head ROI.

Individual data were recorded to a text file, followed by preprocessing in R Software and filtering with a moving average filter of 5 s, as per previous studies^25,36^ (Supplementary Fig 2). The variables evaluated were defined based on Dean et al.’s study, which analyzed head and body movement, and for each ROI of interest were obtained the mean amplitude of motion across the entire video analysis and the coefficient of variability (standard deviation of amplitude of motion divided by mean amplitude of motion across the entire video analysis)^25^. Also, in the base study the total amount of movement was analyzed, however, the videos analyzed here had different durations depending on the individual and, therefore, we used the variable frequency of movements (sum of frames different from zero divided by the total number of frames) to control possible differences due to this. For the analysis, the individual videos were considered (SO and MR).

### Clinical variables

Symptoms were rated on SIPS^55^ and considering the findings of correlation between movements abnormalities and symptoms^19,23,24,36^, we considered for analysis the negative and positive items: social anhedonia (N1), avolition (N2), expression of emotion (N3), experience of emotions and self (N4), ideational richness (N5), unusual thought content (P1), suspiciousness/persecutory ideas (P2) grandiose ideas (P3), perceptual abnormalities/ hallucinations (P4), disorganized communication (P5). To estimate conversion, the SIPS risk calculator (SIPS-RC) was used. The SIPS-RC is a simple calculator that use four predictors based on SIPS items: functional decline, positive symptoms, negative symptoms and general symptoms to provide a solid estimate of conversion outcome^60^. We used the risk path provide in the base article to calculate the individual risk of our sample, in which paths of “yes” or “no” are determined by the severity of these symptoms and lead to a specific risk estimate.

### Statistical analysis

Shapiro-Wilk test was used to evaluate the distribution of the three variables described above (mean amplitude, frequency of movements and coefficient of variability). We used the independent-samples *T* tests to determine whether these variables differed between ARMS and controls. For that, in cases where the distribution was not normal, the non-parametric test Mann–Whitney *U* was performed and for normal distribution the parametric Student’s t-test was used. For the correlation between symptoms obtained by SIPS and movement variables, the continuous and ordinal data were submitted to Kendall’s tau-B correlation coefficient. Given the heterogeneity of our sample, we also evaluate possible impacts of age—using Spearman correlation—and sex—by Kendall’s tau-B correlation—on the motor variables obtained here. Bonferroni correction for multiple comparisons was used eliciting in a significant p-value of <0.0125 for the first set of analysis (ARMS × controls), and of <0.001 for the second set of analysis (symptoms × movement). All statistical tests were performed for each video category in Jamovi 1.8.2. (Windows) and the hypotheses considered were that previously described, that (1) ARMS individuals as compared to controls have lower mean energy motion/lower frequency of movements, (2) that they have a higher variability of movements, and that (3) there is a correlation of these movement variables with negative and positive symptoms.

### Ethics and inclusion statement

All the participants provided written informed consent for this study and its use of data, and the research was approved by the research ethics committees at the Comissão Nacional de Ética em Pesquisa (No. 53536816.0.0000.0065) and Comitê de Ética em Pesquisa da Faculdade de Medicina da Universidade de São Paulo (No. 36510820.3.0000.0068). The research included local researchers in the process of study design, implementation, and data ownership, with outside collaboration only for the writing of the manuscript.

## Supplementary information

Supplementary Figure 1.

Supplementary Figure 2.

## Data Availability

Given the sensitivity of video of individuals faces, video data are not available. However, MEA and demographic variables are available in Excel format. Also given ethical restrictions related to the participants, the data are available under request from the author A.A.L.
