# Supplementary figures and images for: Motion energy analysis during speech tasks in medication-naïve individuals with at-risk mental states for psychosis
Source: Schizophrenia (Heidelb). 2022 Sep 16;8(1):73. doi: 10.1038/s41537-022-00283-3 (PMC9481869; doi:10.1038/s41537-022-00283-3)

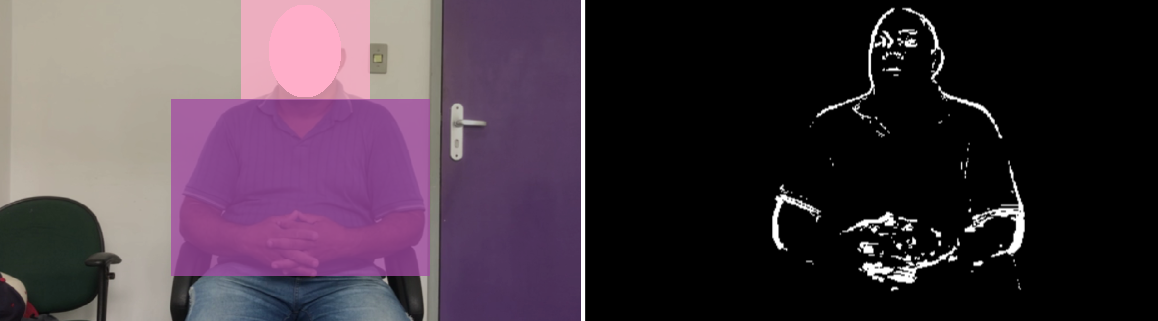

Supplement: Supplementary file 1 — Supplementary Figure 1. [file 41537_2022_283_MOESM1_ESM.png]

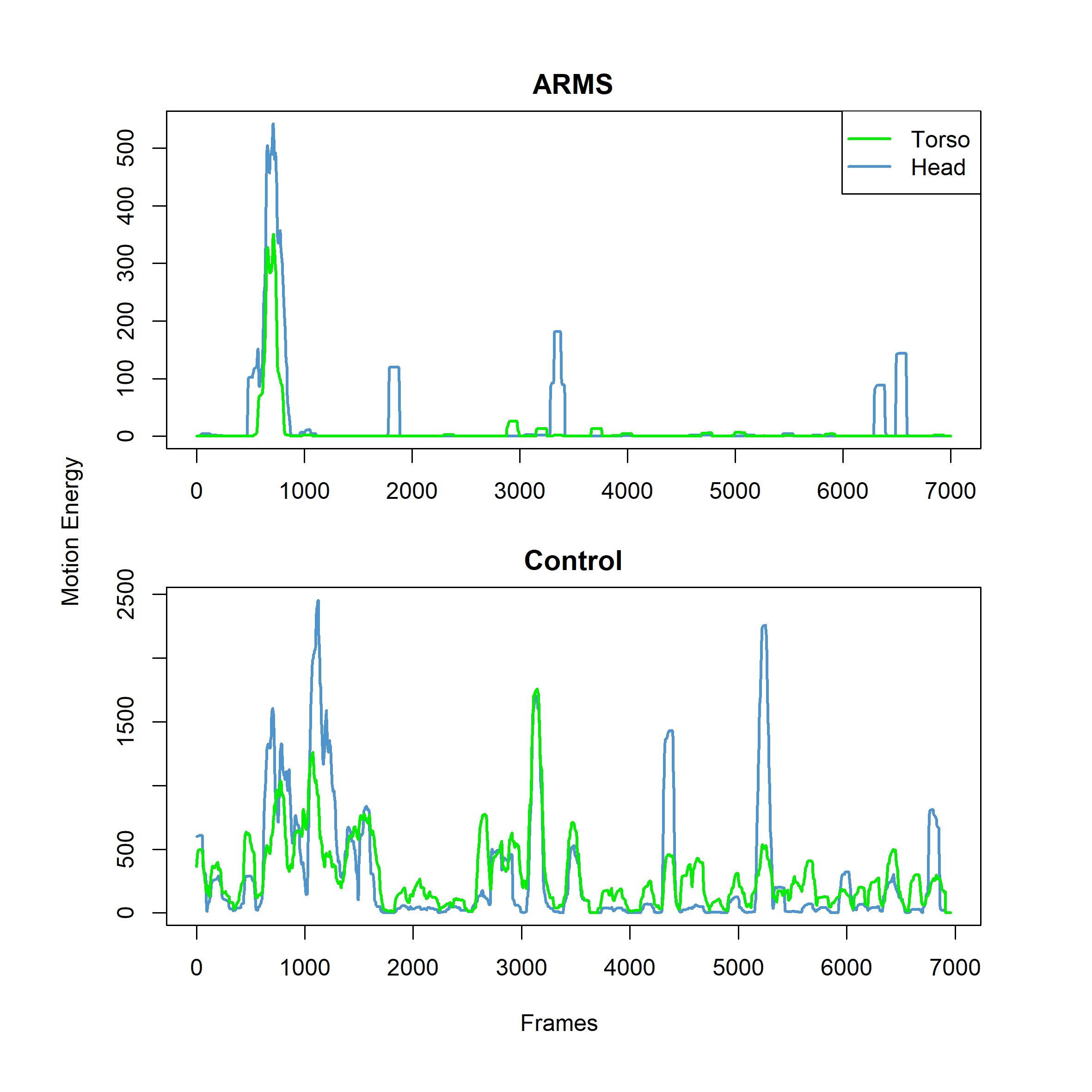

Supplement: Supplementary file 2 — Supplementary Figure 2. [file 41537_2022_283_MOESM2_ESM.png]
